# Secondary cardiac amyloidosis in a patient with mixed connective tissue disease: A case report

**DOI:** 10.1002/ccr3.7669

**Published:** 2023-07-06

**Authors:** Ujjwal Prakash Khanal, Prinska Ghimire, Tejash Shahi, Tulsi Ram Dhakal, Saket Jha

**Affiliations:** ^1^ Institute of Medicine, Tribhuwan University Teaching Hospital Kathmandu Nepal

**Keywords:** amyloidosis, heart failure, mixed connective tissue disease, systemic lupus erythematosus

## Abstract

We report the case of a 62‐year‐old man who presented with shortness of breath, cough, bilateral lower limbs' swelling, and blackish discoloration of multiple fingertips over the past 2 months. Anti‐Ribonucleoprotein antibodies were found to be present, and gadolinium‐based cardiac MRI showed non‐vascular subendocardial enhancement with diffuse symmetrical thickening of the left ventricular wall. A diagnosis of Mixed connective tissue disease with secondary cardiac amyloidosis was thus made, and the patient was successfully managed with intravenous cyclophosphamide, corticosteroids, and other supportive measures. Although extremely rare, this case shows that secondary cardiac amyloidosis should be considered while managing patients with MCTD.

## 1 INTRODUCTION

Mixed connective tissue disease (MCTD) is a rheumatological condition that presents as an overlap between systemic lupus erythematosus (SLE), rheumatoid arthritis (RA), systemic sclerosis (SSc) and polymyositis, characterized by the presence of anti‐U1 small nuclear ribonucleoprotein (anti‐RNP) antibodies.^1^ With a point prevalence ranging from 1.9 to 3.8 per 100,000, this rare condition has shown a clear female preponderance across studies and commonly presents with features of polyarthritis, Raynaud's phenomenon, esophageal hypomobility, and swollen hands.^2^, ^3^, ^4^ Cardiac involvement is not uncommon, and is usually seen in the form of myocarditis, arrhythmias, or coronary vessel disease.^5^

While the risk for secondary amyloidosis (SA) has been clearly demonstrated in other inflammatory conditions like RA, there are extremely few reports of SA in MCTD.^6^, ^7^ Furthermore, cardiac involvement due to SA in a patient with MCTD has not been reported yet.

Here, we present the case of a 62‐year‐old man diagnosed with MCTD and concurrent secondary cardiac amyloidosis. To our knowledge, this is the first report of this condition in published literature.

## 2 CASE REPORT

In February 2022, A 62‐year‐old man of Indo‐Aryan ethnicity presented to the emergency room with complaints of shortness of breath and cough for 2 months, which had worsened in the past week. The shortness of breath was insidious in onset, initially starting as Modified Medical Research Council (MMRC) grade I, but had gradually progressed to MMRC grade IV over the course of illness. This was associated with productive cough with scanty amounts of mucoid sputum, and swelling of both lower limbs for the same duration. Additionally, he complained of a tingling sensation followed by blackish discoloration in multiple fingertips of both hands over the past 2 weeks, which was associated with a continuous, throbbing pain not relieved by over‐the‐counter analgesics. He also complained of dry eyes and dry mouth, but there was no history of skin/oral ulcers, rashes, or hair loss. There was no history of chest pain, palpitations, or fainting attacks. The patient also denied any night sweats or weight loss. He was an active smoker with a smoking history of 20 pack years, having quit a year ago. The family history was insignificant.

On examination, his heart rate was 136 beats per minute, blood pressure was 110/70 mmHg, respiratory rate was 20 breaths per minute, and he was afebrile. He had bilateral pitting edema of the feet and raised jugular venous pressure (JVP), but with no palpable hepatomegaly. On chest auscultation, there was decreased air entry with crepitations in bilateral basal regions. No murmurs were heard. Examination of hands revealed bluish‐black discoloration of distal phalanges of multiple fingers which appeared swollen and sausage‐like. The areas were cold and severely tender on palpation without any blistering of skin or other gross deformities. Other systemic examinations were unremarkable. With these findings, he was referred to us for rheumatological consultation.

Upon investigation, blood counts were consistently within normal range. Serum electrolytes, renal function parameters, lipid profile, and liver function parameters were all normal except for a decreased total protein (5.37 g/dL) and hypoalbuminemia (2.77 g/dL). 24‐h‐urine protein was 0.46 g, and the 24‐h‐urine creatinine was lower than normal. His ESR was normal, but the CRP was elevated to 56.4 mg/L. His serum LDH was 743 U/L (125‐220 U/L), D‐dimer was 1.19 mg/L (<0.5 mg/L), procalcitonin was 0.17 ng/mL (<0.5 ng/mL), CPK was 231.3 U/L (55‐170 U/L), but troponin I was normal. His NT‐ProBNP level was significantly raised to 5740 pg/mL (<270 pg/mL).

Ultrasound of the abdomen revealed interloop ascites and a chest x‐Ray showed bilateral pleural effusion. Considering the prolonged history of cough and shortness of breath, sputum and pleural fluid were sent for analysis, which were repeatedly negative for bacterial growth, acid fast bacilli, or malignant cells. The CECT scan of chest and abdomen demonstrated air space consolidation in central peribronchovascular distribution with diffuse and bilateral perilymphatic interlobular septal thickening, suggestive of pulmonary edema, along with moderate bilateral pleural effusions with adjacent collapse/consolidation. HRCT of the chest further showed air space patchy opacities with peribronchovascular thickening in multiple lobes of both lungs, suggesting acute interstitial pulmonary edema with superimposed infectious process, likely pneumonia.

His ECG readings were persistently normal, but his echocardiography demonstrated features of restrictive cardiomyopathy with moderate concentric left ventricular hypertrophy, grade II left ventricular (LV) diastolic dysfunction with raised LV filling pressure, normal LV systolic function (LVEF = 70%), minimal pericardial effusion, and no regional wall motion abnormalities. Cardiac MRI showed diffuse symmetrical thickening of left ventricular wall with preserved LVEF of 78%, along with diffuse non‐vascular subendocardial and mild myocardial enhancement in the LV myocardium in delayed gadolinium (GAD) images, suggestive of cardiac amyloidosis.

A series of investigations were simultaneously performed to explore digital gangrene. Venous and arterial doppler study of bilateral upper limbs revealed gradual decrease in velocities of bilateral ulnar arteries, increased velocities of digital and palmar arteries, and focal dilatations of proximal forearm and wrist veins. Rheumatology profile demonstrated the presence of U1 SM/RNP antibodies (strong positive) and Smith antibodies (positive). His serum was negative for other autoimmune markers, namely ANA (anti‐DNP), anti‐CCP, p‐ANCA, c‐ANCA, SS‐A, RO‐52, SS‐B, anti‐histones, anti‐centromere, SCL‐70 IgG, PM‐SCL, PCNA, nucleosome, AMA‐M2, and ribosomal P antibodies, with normal levels of C3, and C4. His serum was also negative for lupus anticoagulant, anticardiolipin antibodies (IgG and IgM), beta2 glycoprotein antibodies (IgG and IgM), and anti DsDNA antibodies.

With these findings, a diagnosis of MCTD with heart failure with preserved LVEF secondary to cardiac amyloidosis with digital gangrene was made. Treatment with immunosuppressants was started, with the first cycle of cyclophosphamide at 10 mg/kg, along with a tapering dose of oral steroids. Additional management was done with diuretics, ivabradine, anticoagulants, pregabalin, tadalafil, and other supportive measures. After the first cycle, significant improvement was observed with decrease in pain and tingling sensation in the fingers as well as disappearance of pedal edema and shortness of breath, with no further progression of gangrene. A total of 6 cycles of cyclophosphamide was administered, with an interval of at least 3 weeks between 2 cycles. During the course of treatment, no significant adverse effects were noted on routine clinical and lab monitoring, and the improvement was found to be maintained at 3 month follow‐up.

## 3 DISCUSSION

MCTD was first described in 1972 by Sharp et al. as a distinct rheumatological condition presenting with features of SLE, scleroderma and polymyositis, characterized by the presence of anti‐RNP antibodies, and showing excellent response to corticosteroids.^1^ Since the concept was first introduced, it has been met with significant criticism, largely because many features heavily correspond with other inflammatory rheumatological conditions, and many patients (including the ones originally reported in 1972) often meet the criteria for SLE or RA with time.^8^, ^9^ Nevertheless, several other studies have supported the existence of MCTD as a unique condition, a big proponent for which has been the association of anti‐RNP antibodies with HLA‐DR4 haplotype and the relative specificity of the U1‐70k subtype of anti‐RNP antibodies with MCTD.^10^, ^11^

A nationwide study performed in 2011 in Norway estimated the incidence of MCTD at 2.1 per million per year. The study also highlighted a clear female preponderance with a female‐to‐male ratio of 3.3:1, which has been supported by other studies.^2^, ^3^ Since its inception, however, at least four sets of criteria have been proposed for diagnosing MCTD, and this lack of a unified system has created discrepancies in reported incidence worldwide.^2^, ^12^ The simplest and the most popular criteria were proposed by Alarcon‐Segovia, which requires serological detection of anti‐RNP antibodies with at least three clinical criteria (including edema in hands, myositis, Raynaud's phenomenon, and acrosclerosis). Other studies performed since have consistently shown that polyarthritis, Raynaud's phenomenon, puffy fingers, esophageal dysmotility, and sclerodactyly are the commonest symptoms, while cardiovascular thrombotic events and pulmonary hypertension are less common but grave complications.^4^ This corresponds to our findings, where the patient presented with features of polyarthritis, swollen fingers, and Raynaud's phenomenon. However, secondary cardiac amyloidosis seen in our patient has never been reported in published literature.

Systemic amyloidoses are a rare group of conditions characterized by deposition of insoluble “amyloid” proteins in the extracellular space of various organs. Although known to originate from more than 30 different types of proteins in the body, these misfolded proteins share a remarkably similar structure with beta‐strands forming pleated sheets that are resistant to normal proteolysis.^13^ This results in infiltrative lesions in the kidneys, heart, nervous system, blood vessels, and other soft tissues.^14^ Cardiac involvement in particular is challenging to identify and treat.^15^

Cardiac amyloidosis can occur as a part of primary (AL) amyloidosis, where immunoglobulin light chains produced by defective plasma cells form amyloid deposits. Sixty percent of all patients with this variant show cardiac involvement, but it is not commonly encountered owing to the rarity of the disease, and associated poor prognosis.^16^ Much more common, however, are the senile and secondary (AA) forms that occur in response to age and other inflammatory conditions like RA or inflammatory bowel disease (IBD). These inflammatory conditions produce a sustained acute phase response leading to overproduction (and thus misfolding) of serum amyloid A (SAA) proteins.^17^ Although this particular sequence has never been described in a patient with MCTD, considering the close pathophysiological nature of MCTD with other rheumatological conditions, it can be assumed that there is a similar underlying pathology.

Whatever the underlying mechanism, cardiac amyloidosis is characterized by infiltration of amyloid nodules and fibrils between the myocytes, disrupting both the contractile and electrical pathways of the heart. Additionally, amyloid deposits in and around arterioles have been shown to disrupt coronary flow.^16^, ^17^ All these can lead to biventricular wall thickening and subsequent restrictive cardiac failure, which mostly manifests as bilateral pedal edema, ascites, hepatomegaly, and raised JVP.^18^ There have also been several reports of myocardial ischemia or angina in these patients.^19^, ^20^ It is interesting to note, however, that these features are not very pronounced in patients with secondary cardiac amyloidosis, making our case particularly unique.^21^

Although non‐invasive criteria have been proposed for other forms of amyloidosis, the gold standard for diagnosing cardiac amyloidosis remains biopsy with demonstration of amorphous pink deposits on congo‐red staining under light microscopy and apple green birefringence under polarized light.^16^, ^22^ Rise in serum troponin I and brain natriuretic peptides (BNP) have also been shown to be present, though their role in diagnosis and monitoring has not yet been established.^23^, ^24^ The ECG findings are more reliable, showing pseudo‐infarction patterns, low‐voltage QRS amplitudes and varying degrees of conduction delays, reflective of ventricular wall thickening and amyloid infiltration in the conduction pathways.^18^ An echocardiogram can verify these findings further, showing biventricular increase in wall thickness, absence of ventricular dilation, LV diastolic dysfunction, atrial septal thickening, and a pathognomonic “granular sparkling” pattern.^18^ The ECG and echocardiography findings can help establish an infiltrative pathology, whereas MRI findings with diffuse sub‐endocardial late gadolinium enhancement can effectively distinguish amyloidosis from other causes of restrictive cardiomyopathy even in the absence of a biopsy.^25^

Our patient had presented with blackish discoloration and pain in fingers, along with tingling sensations and severe tenderness on examination, which was further associated with dry eyes and dry mouth. This led to the suspicion of a rheumatological disorder, and a diagnosis of MCTD was made based on clinical features and the presence of U1 SM/RNP antibodies in the absence of other autoimmune biomarkers. Similarly, clinical features of bilateral pitting edema, distension of abdomen, and shortness of breath led to suspicion of cardiac involvement, despite previous diagnosis of pneumonia. Echocardiography demonstrated findings corresponding with restrictive cardiomyopathy. Although cardiac biopsy was not performed owing to resource and technological constraints, a diagnosis of cardiac amyloidosis could be made based on MRI findings. While a definitive causal relationship between MCTD and amyloidosis cannot be established, the amyloidosis was deemed secondary in our patient because the natural history did not fit the aggressive profile associated with primary form of amyloidosis, and the patient's age made senile amyloidosis unlikely. This was further supported by the history, the presence of distinct rheumatological markers for MCTD, and a lack of another plausible explanation for the amyloid deposition despite extensive diagnostic effort.

Despite this being the first report of cardiac amyloidosis in a patient with MCTD, there have been a few other reports of non‐cardiac SA in patients with MCTD.^7^, ^26^, ^27^ Their reports have been largely similar to ours, but it is notable that features of cardiac dysfunction occurred in our patient before typical rheumatological symptoms were seen, whereas previous cases report amyloidosis appearing years, if not decades, after the beginning of the original rheumatological disease. We theorize that there may have been subclinical inflammation induced by MCTD with a prolonged acute phase response in our patient, leading to relatively synchronous presentation of both MCTD and amyloidosis.

As of now, there are no established treatment guidelines for MCTD, so the therapy administered is often guided by the symptomatology and the specific organ systems involved. Although the original description of MCTD by Sharp et al. mentioned good steroid response as a principal feature of the disease, other studies have shown that most MCTD patients, despite responding well to steroids for inflammatory manifestations like arthritis and serositis, often require additional cytotoxic therapy, especially in presence of cutaneous or sclerodermatous manifestations.^5^, ^28^, ^29^ Cardiac amyloidosis, on the other hand, does not have any specific treatment modalities available. The best options are to control the primary inflammatory disease, perform careful diuresis if needed, and to install a pacemaker to correct rhythm abnormalities if indicated.^16^ Considering organ threatening cutaneous manifestation (digital gangrene) in our patient, we started treatment with cyclophosphamide and oral steroids, based on prior evidence as well as our previous experiences of halting organ‐threatening rheumatological manifestations with cyclophosphamide. This led to excellent symptomatic improvement with no serious side effects.

## 4 CONCLUSION

MCTD may present with features of concurrent cardiac amyloidosis, requiring clinicians to understand the distinct (and often difficult) pathophysiological mechanisms and diagnostic principles behind the two diseases. Although little is reported about both these conditions and their management, our experience of managing the case with cyclophosphamide, steroids, and other supportive measures led to excellent symptomatic recovery. Further research and larger case series are required to understand details about the disease presentation, diagnosis, and potential treatment methods.

## AUTHOR CONTRIBUTIONS

**Ujjwal Prakash Khanal:** Data curation; writing – original draft; writing – review and editing. **Prinska Ghimire:** Data curation; writing – original draft; writing – review and editing. **Tejash Shahi:** Data curation; writing – original draft; writing – review and editing. **Tulsi Ram Dhakal:** Data curation; writing – original draft. **Saket Jha:** Conceptualization; supervision; writing – review and editing.

## FUNDING INFORMATION

No funding was required in the preparation of this case report.

## ETHICS STATEMENT

This study is in compliance with the declaration of Helsinki.

## CONSENT

Written informed consent was obtained from the patient for publication of this case report in accordance with the journal's patient consent policy.

## Data Availability

All data generated or analyzed during this study are included in this published article.

## References

[1] Sharp GC , Irvin WS , Tan EM , Gould RG , Holman HR . Mixed connective tissue disease‐an apparently distinct rheumatic disease syndrome associated with a specific antibody to an extractable nuclear antigen (ENA). Am J Med. 1972;52(2):148‐159. 4621694 10.1016/0002-9343(72)90064-2

[2] Ungprasert P , Crowson CS , Chowdhary VR , Ernste FC , Moder KG , Matteson EL . Epidemiology of mixed connective tissue disease, 1985‐2014: a population‐based study. Arthritis Care Res. 2016;68(12):1843‐1848. 10.1002/acr.22872 PMC5426802 26946215

[3] Gunnarsson R , Molberg O , Gilboe IM , Gran JT . The prevalence and incidence of mixed connective tissue disease: a national multicentre survey of Norwegian patients. Ann Rheum Dis. 2011;70(6):1047‐1051. 21398332 10.1136/ard.2010.143792

[4] Tani C , Carli L , Vagnani S , et al. The diagnosis and classification of mixed connective tissue disease. J Autoimmun. 2014;48‐49:46‐49. 10.1016/j.jaut.2014.01.008 24461387

[5] Hajas A , Szodoray P , Nakken B , et al. Clinical course, prognosis, and causes of death in mixed connective tissue disease. J Rheumatol. 2013;40(7):1134‐1142. 23637328 10.3899/jrheum.121272

[6] Zhou J , Dai Y , Lin Y , Chen K . Association between serum amyloid a and rheumatoid arthritis: a systematic review and meta‐analysis. Semin Arthritis Rheum. 2022;52:151943. 35027248 10.1016/j.semarthrit.2021.12.011

[7] Kimura H , Komatsuda A , Sawada K , Mimori A , Baba S , Minota S . Rapidly progressed secondary amyloidosis in a patient with mixed connective tissue disease. Intern Med. 2004;43(9):878‐882. 15497530 10.2169/internalmedicine.43.878

[8] Zimmermann C , Steiner G , Skriner K , Hassfeld W , Petera P , Smolen JS . The concurrence of rheumatoid arthritis and limited systemic sclerosis: clinical and serologic characteristics of an overlap syndrome. Arthritis Rheum. 1998;41(11):1938‐1945. 9811047 10.1002/1529-0131(199811)41:11<1938::AID-ART7>3.0.CO;2-X

[9] Nimelstein SH , Brody S , McShane D , Holman HR . Mixed connective tissue disease: a subsequent evaluation of the original 25 patients. Medicine. 1980;59(4):239‐248. 6967141

[10] Ruuska P , Hämeenkorpi R , Forsberg S , et al. Differences in HLA antigens between patients with mixed connective tissue disease and systemic lupus erythematosus. Ann Rheum Dis. 1992;51(1):52‐55. 1540038 10.1136/ard.51.1.52 PMC1004618

[11] Habets WJ , de Rooij DJ , Salden MH , et al. Antibodies against distinct nuclear matrix proteins are characteristic for mixed connective tissue disease. Clin Exp Immunol. 1983;54(1):265‐276. 6352106 PMC1536194

[12] Amigues JM , Cantagrel A , Abbal M , Mazieres B . Comparative study of 4 diagnosis criteria sets for mixed connective tissue disease in patients with anti‐RNP antibodies. Autoimmunity Group of the Hospitals of Toulouse. J Rheumatol. 1996;23(12):2055‐2062. 8970041

[13] Wechalekar AD , Gillmore JD , Hawkins PN . Systemic amyloidosis. Lancet. 2016;387(10038):2641‐2654. 26719234 10.1016/S0140-6736(15)01274-X

[14] Cohen AS . Amyloidosis. N Engl J Med. 1967;277(10):522‐530. 5340627 10.1056/NEJM196709072771006

[15] Martinez‐Naharro A , Hawkins PN , Fontana M . Cardiac amyloidosis. Clin Med. 2018;18(Suppl 2):s30‐s35. 10.7861/clinmedicine.18-2s-s30 PMC6334035 29700090

[16] Shah KB , Inoue Y , Mehra MR . Amyloidosis and the heart: a comprehensive review. Arch Intern Med. 2006;166(17):1805‐1813. 17000935 10.1001/archinte.166.17.1805

[17] Scarpioni R , Ricardi M , Albertazzi V . Secondary amyloidosis in autoinflammatory diseases and the role of inflammation in renal damage. World J Nephrol. 2016;5(1):66‐75. 26788465 10.5527/wjn.v5.i1.66 PMC4707170

[18] Dubrey SW , Cha K , Anderson J , et al. The clinical features of immunoglobulin light‐chain (AL) amyloidosis with heart involvement. QJM. 1998;91(2):141‐157. 9578896 10.1093/qjmed/91.2.141

[19] Ishikawa Y , Ishii T , Masuda S , et al. Myocardial ischemia due to vascular systemic amyloidosis: a quantitative analysis of autopsy findings on stenosis of the intramural coronary arteries. Pathol Int. 1996;46(3):189‐194. 10846569 10.1111/j.1440-1827.1996.tb03597.x

[20] Saffitz JE , Sazama K , Roberts WC . Amyloidosis limited to small arteries causing angina pectoris and sudden death. Am J Cardiol. 1983;51(7):1234‐1235. 6837470 10.1016/0002-9149(83)90379-x

[21] Gillmore JD , Lovat LB , Persey MR , Pepys MB , Hawkins PN . Amyloid load and clinical outcome in AA amyloidosis in relation to circulating concentration of serum amyloid a protein. Lancet. 2001;358(9275):24‐29. 11454373 10.1016/S0140-6736(00)05252-1

[22] Garcia‐Pavia P , Rapezzi C , Adler Y , et al. Diagnosis and treatment of cardiac amyloidosis: a position statement of the ESC Working Group On Myocardial And Pericardial Diseases. Eur Heart J. 2021;42(16):1554‐1568. 33825853 10.1093/eurheartj/ehab072 PMC8060056

[23] Takemura G , Takatsu Y , Doyama K , et al. Expression of atrial and brain natriuretic peptides and their genes in hearts of patients with cardiac amyloidosis. J Am Coll Cardiol. 1998;31(4):754‐765. 9525543 10.1016/s0735-1097(98)00045-x

[24] Miller WL , Wright RS , McGregor CG , et al. Troponin levels in patients with amyloid cardiomyopathy undergoing cardiac transplantation. Am J Cardiol. 2001;88(7):813‐815. 11589859 10.1016/s0002-9149(01)01877-x

[25] Siddiqi OK , Ruberg FL . Cardiac amyloidosis: an update on pathophysiology, diagnosis, and treatment. Trends Cardiovasc Med. 2018;28(1):10‐21. 28739313 10.1016/j.tcm.2017.07.004 PMC5741539

[26] Piirainen HI , Helve AT , Törnroth T , Pettersson TE . Amyloidosis in mixed connective tissue disease. Scand J Rheumatol. 1989;18(3):165‐168. 2772564 10.3109/03009748909095415

[27] Lee CP , Choong C , Eu KM , Tee KS , Abisheganaden J . A case of nodular pulmonary amyloidosis in a patient with mixed connective tissue disease (MCTD). Chest. 2016;149(4):A195.

[28] Cappelli S , Bellando Randone S , Martinović D , et al. "To be or not to be," ten years after: evidence for mixed connective tissue disease as a distinct entity. Semin Arthritis Rheum. 2012;41(4):589‐598. 21959290 10.1016/j.semarthrit.2011.07.010

[29] Chen KR , Carlson JA . Clinical approach to cutaneous vasculitis. Am J Clin Dermatol. 2008;9(2):71‐92. 18284262 10.2165/00128071-200809020-00001

